# The efficacy of probiotics in management of recurrent aphthous stomatitis: a systematic review and meta-analysis

**DOI:** 10.1038/s41598-020-78281-7

**Published:** 2020-12-03

**Authors:** Bin Cheng, Xinyi Zeng, Shaoyuan Liu, Jing Zou, Yan Wang

**Affiliations:** State Key Laboratory of Oral Diseases, National Clinical Research Center for Oral Diseases and Department of Pediatric Dentistry, West China Hospital of Stomatology, Sichuan University, Chengdu, 610041 China

**Keywords:** Diseases, Medical research

## Abstract

There is currently a lack of effective drugs to cure recurrent aphthous stomatitis. This study aimed to evaluate the efficacy of probiotics alone or as an adjunct in recurrent aphthous stomatitis (RAS) patients. Seven randomized controlled trials (RCTs) were included, of which three were included in quantitative analysis. Of five studies evaluating the efficacy of probiotics alone compared with placebo or Oracure gel, two reported no significant difference in relieving oral pain, while probiotics exhibited a higher capacity for decreasing oral pain in the other three. A significant decrease in ulcer severity was found in one, while no significant difference was found in the other four. The remaining two studies demonstrated that probiotics, as an adjunct to steroids or anaesthetic antiseptic gel, significantly reduced the ulcer severity and oral pain. The meta-analysis showed a significant decrease in oral pain (− 1.72, P = 0.0001) with probiotics compared with placebo. In conclusion, probiotics alone were capable of relieving oral pain but not effective in reducing ulcer severity. A combination of probiotics and steroids or anaesthetic antiseptic gel was more effective than steroids or anaesthetic antiseptic gel alone in RAS patients. Probiotics are promising for the treatment of recurrent aphthous stomatitis.

## Introduction

Recurrent aphthous stomatitis (RAS) is the most common self-limited oral mucosal disease worldwide, with a prevalence ranging from 5 to 66% in different countries^1,2^. RAS is characterized by recurrent round or elliptical ulcers that can occur anywhere in the oral cavity and are common in the non-keratinized epithelium, such as the lingual margin and buccal and labial mucosa^3^. Before the condition breaks out, the ulcer site often has a burning sensation for 2–48 h^4^. Lesions are often single but are sometimes multiple. The ulcer site is often covered with a yellow or gray pseudomembrane, with hyperemia in the surrounding mucosa^5–7^. RAS could cause severe oral pain and affect swallowing and chewing, adversely affecting the quality of life (QoL).

Although the etiology of RAS is still unclear, many studies have demonstrated that innate immunity and adaptive immunity have an essential role in the development of RAS. L-type bacteria isolated from the aphthous ulcerations might play a role in the occurrence of oral ulcerations^8^. Abnormal growth of the oral microbial flora might be the cause of RAS, and several microorganisms related to the ulcers have been identified^9^. Besides, microbiological factors were also reported to modify the immune response and cause RAS^10^.

Current treatment for RAS is primarily symptomatic, aiming at alleviating pain, promoting ulcer healing, and trying to prevent a recurrence. However, no therapy can guarantee a definitive cure. Topical treatment and systemic therapy are the most common treatment methods. Interventional methods recommended for topical treatment include glucocorticoids, anti-inflammatory drugs, analgesics, antibiotics, antiseptics, and low-level lasers^11^. Therapeutic agents proposed for systemic use include immunosuppressants, immunomodulators, and immunopotentiators^12^. However, traditional treatments have many limitations. For example, when the ulcer is deep and large, the efficacy of topical treatment is reduced. The long-term application of steroids, the most common drug in the treatment of RAS, might give rise to serious adverse events, including oral mucosa atrophy and immunity deficiency^13^. The side effects of systematic glucocorticoids are too severe for many people to bear. Therefore, it is necessary to develop new therapies with higher efficiency and lower side effects.

Probiotics, a healthy component of the normal oral microbial community, are capable of promoting the growth of other beneficial microorganisms and modulating host immune responses^3,14^. The “probiotics” concept was first introduced in 1965 as an antonym for antibiotics by Lilly and Stillwell. Probiotics would benefit the host if administered in sufficient amounts^15^. Probiotics play an essential role in systemic health, affecting the immune system and the host’s intestinal epithelium to regulate the function of corresponding organs^16^. Furthermore, probiotics (mainly *Lactobacilli* and *Bifidobacteria*) have also been introduced to oral medicine in recent years. Many studies have already assessed the efficacy of probiotics in preventing caries, gingivitis, cancer therapy-induced oral mucositis, periodontitis, and peri-implant diseases, which are related to bacterial biofilm and host immune response. The results support the potential use of probiotics in managing gingivitis, cancer therapy-induced oral mucositis, and periodontitis^17–20^.

As the RAS is associated with bacterial factors and host immune reactions, probiotics have been suggested to have the potential to treat RAS. In 2012, RAS was successfully managed by *Lactobacillus brevis* CD2 in an immunocompromised hemophilic patient after the failed attempt to use antiviral agents and topical steroids; the condition did not recur for 6 months^19^. And probiotics were proposed as a treatment for oral aphthous stomatitis in patients with inflammatory bowel disease^21^.

In recent years, some clinical studies have explored the efficacy of probiotics in decreasing ulcer severity and alleviating oral pain in RAS patients. However, due to the conflicting results, the efficacy of probiotics is still controversial. Thus, this systematic review and meta-analysis aimed to investigate the effects of probiotics alone or as an adjunctive treatment on RAS patients.

## Results

### Literature search

**Figure 1:**
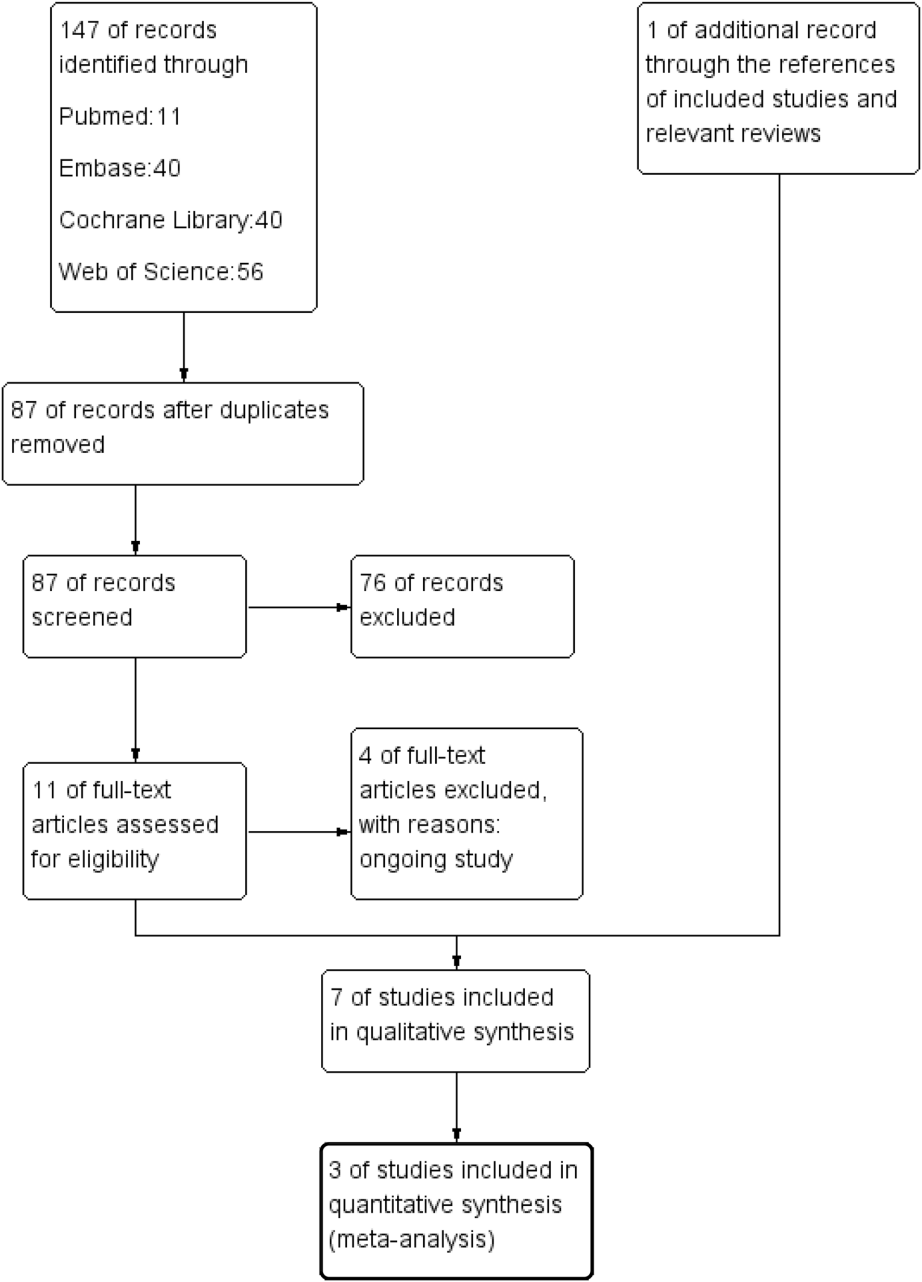
Study selection flowchart for the systematic review.

Figure 1 presents the search strategy outline in a flow diagram. In the initial search, 147 articles were retrieved from the databases, 87 of which remained after eliminating duplicates. After two independent authors (L.S.Y. and C.B.) read the titles and abstracts, eleven studies remained, which were read in full text. six of the eleven studies fulfilled the criteria in the review and were subjected to data extraction. One additional study was found by conducting a manual search of the reference lists. Finally, seven studies were included in the systematic review for qualitative analysis, of which three studies were included in the quantitative analysis.

### Description of the included studies

Table 1 summarizes the characteristics of all the included studies. Seven studies were published between 2011 and 2020, of which two studies published in 2020 and two in 2017, and the other three were published in 2011, 2014, and 2019 respectively. All the seven included studies were randomized controlled trials. The sample size ranged from 19 to 80 participants. Four studies^22–25^ described the age range of the patients, ranging from 8 to 60 years, two studies^26,27^ recorded the mean age that was 55.9, 28.2 in the test group and 36.4, 29.38 in the control group respectively, while the one^28^ did not mention. Aswath et al. (2014)^28^ did not report the gender distribution of the subjects. Three studies^23,25,26^ reported the drop-out numbers as 1, 4, and 15, respectively, but there were none in the four other studies^22,24,27,28^.

**Table 1:** Characteristics of included studies.

Study ID	Study design	No. (M/F)	Mean age (SD, range)	Participants	Interventions	Comparison	Drop-out
Dugourd 2020^26^	RCT	T:10 (4/6) C:9 (4/5)	T:55.9 (17.5, NR) C:36.4 (12.4, NR)	RAS	T (N = 10): probiotic (1.5 billion *Lactobacillus rhamnosus* Lcr35) four times daily for 90 days	C (N = 9):placebo	4^a^
Trinchieri 2011^22^	RCT	T:15 (7/8) C:15 (8/7)	T:NR (NR, 8–36) C:NR (NR, 10–35)	RAS	T (N = 15): 1 lozenges containing *Lactobacillus brevis* CD2 (at least 1 billion live bacteria per lozenge) 4 times daily for 7 days	C (N = 15):placebo	0
Pedersen 2019^25^	RCT	T:10 (2/8) C:10 (5/5)	T:22.7 (NR, 18–28) C:24.8 (NR, 22–30)	RAS	T (N = 10): 1 lozenges (*Lactobacillus reuteri* DSM17938 and ATCC PTA5289, with at least 0.5 billion live bacteria of each strain per lozenge) twice daily for 90 days	C (N = 10): placebo	1^b^
Nirmala 2017^24^	RCT	80 (49/31)	33.7 (1.74, 20–60)	T_1_&C_1_: RAS T_2_&C_2_: oral candidiasis	T_1_ (N = 20) & T_2_ (N = 20): *Bacillus Clausii* probiotic + triamicinolone paste twice daily for 1 week	C_1_ (N = 20) and C_2_ (N = 20): triamicinolone paste twice daily for 1 week	0
Mimura 2017^23^	RCT	T:22 (14/8) C_1_:23 (15/8) C_2_:30 (14/16)	T:36.6 (NR, 19–57) C_1_:38.3 (NR, 23–58) C_2_:37.4 (NR, 23–58)	T&C_1_: RAS C_2_: healthy individuals	T (N = 30): one sachet (1–10 billion CFU *Lactobacillus paracasei* Lpc-37 SD 5275, 1–10 billion CFU *Lactobacillus rhamnosus* HN001 SD 5675, 1–10 billion CFU *Lactobacillus acidophilus* NCFM SD 5221, 1–10 billion CFU *Bifidobacterium lactis* HN019 SD 5674) twice a day for 120 days	C_1_ (N = 30): placebo C_2_ (N = 30):without any treatment	15^c^
Aggour 2020^27^	RCT	T:30 (11/19) C:30 (10/20)	T:28.2 (8.14) C:29.38 (9.06)	Minor RAS	T (N = 30):1 lozenge(*L. acidophilus* 1.5 billion cfu, *Bifidobacterium* *lactis* 1.5 billion cfu) twice daily for 5 days	C (N = 30): Oracure gel twice daily for 5 days	0
Aswath 2014^28^	RCT	T:25 (NR) C:25 (NR)	NR	Minor RAS	T (N = 25): anaesthetic antiseptic gel i.e., mucopain gel and cap. Becosules once a day along with bifilac lozenge(30 million *Streptococcus* *faecalis* T-110, 2 million *Clostridium* *butyricum* TO-A, 1 million *Bacillus* *mesentericus* TO-A, 50 million *Lactobacillus* sporogenes) 3 times a day	C (N = 25): anaesthetic antiseptic gel i.e., mucopain gel and cap. Becosules once a day	0

*No.* number, *M* man, *F* female, *SD* standard deviation, *RCT* randomized controlled trial, *NR* not reported, *T* test group, *C* control group, *CFU* Colony Forming Units, *VAS* Visual Analogue Pain Scale, *USS* Ulcer Severity Score, *OHIP-14* Oral Health Impact Profile 14 questionnaire. ^a^1 refused to continue and treatment never started, 1 screen failure and treatment never started, 1 lost to follow-up, 1 refused to continue. ^b^Data from day 90 is missing in 1 patient in the test group. ^c^The author didn’t mention the reason that why those participants dropped out.

Nirmala et al. (2017)^24^ assigned patients to the AU and BU groups for recurrent aphthous ulcers. Patients were treated with oral probiotics as an adjunct to triamcinolone in the AU group and with a triamcinolone paste in the AC group. Aswath et al. (2014)^28^ advised patients in test group take anaesthetic antiseptic gel along with bifilac lozenge composed of probiotics while patients in control group take anaesthetic antiseptic gel only. In the five other studies, the intervention in the experimental group consisted of probiotics only, while the control group subjects received a placebo^22,24–27^. Patients in studies by Mimura et al. (2017)^23^ and Aswath et al. (2014)^28^ underwent a symbiotic treatment combined with four probiotics, and Pedersen et al. (2019)^25^ gave patients a combination of two. Some other studies adopted a single probiotic treatment^22,24,26,27^. Concerning the outcome measures, the evaluation assessment for the severity of ulcers are diverse and will be described in detail later. Three studies^23,25,26^ using the visual analog (pain) scale (VAS) for the evaluation of oral pain were included in the meta-analysis; four studies^22,24,27,28^ were qualitatively described.

### Quality of the included studies

**Figure 2:**
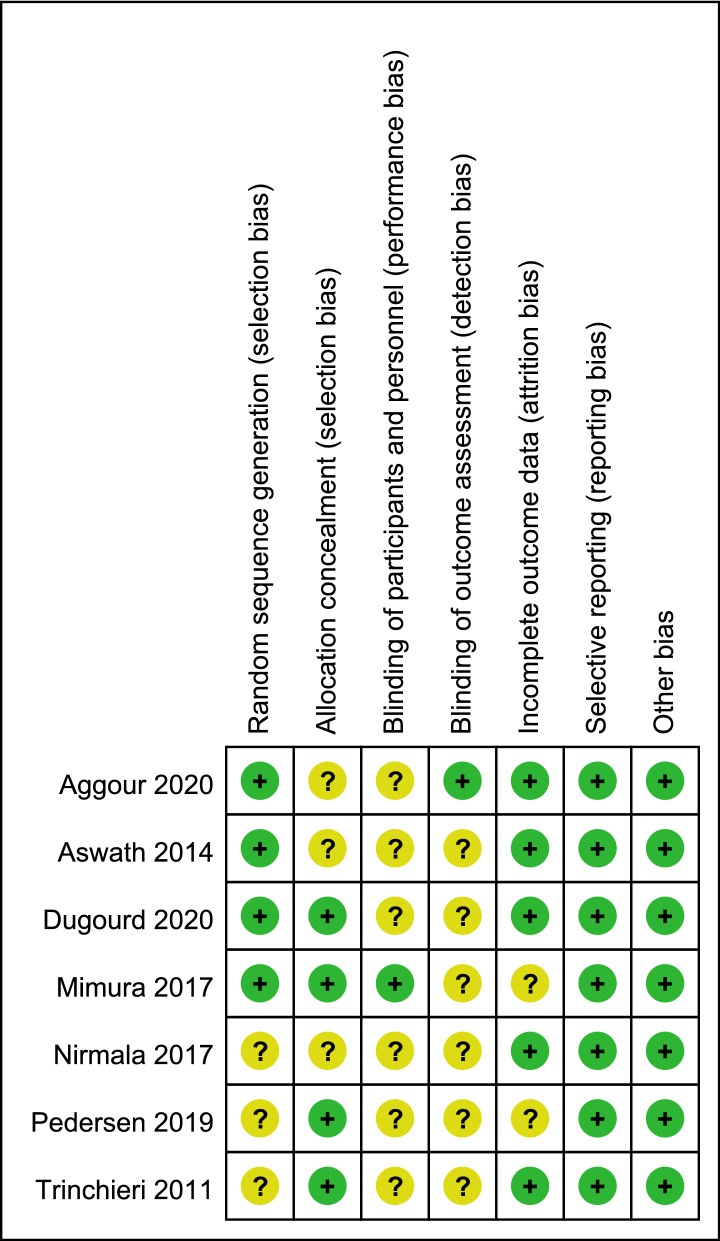
Risk of bias summary: review authors’ judgment about each risk of bias item for each included study.

The Cochrane Collaboration’s tools for assessing the risk of bias and study quality were used to assess the quality of research. Figure 2 presents the detailed results of the assessment. All the seven studies were unclear. The method for generating random sequences in three studies was unclear (Nirmala et al., 2017; Pederson et al., 2019; Trinchieri et al., 2011)^22,24,25^. Three studies (Nirmala et al., 2017; Aggour et al., 2020; Aswath et al., 2014) did not explain the method of allocation concealment^24,27,28^. When it comes to the blinding of participants and personnel, Mimura et al. 2017 was low and the other six unclear. Moreover, only Aggour et al. (2020)^27^ mentioned that the study outcome was assessed using blinding methods^27^. Mimura et al. (2017)^23^ and Pederson et al. (2019)^25^ did not present complete outcomes. The risks of selective reporting and other risks were low in all studies. Of all the studies, Nirmala et al. (2017)^24^ and Pederson et al. (2019)^25^ had four unclear points, and Trinchieri et al. (2011)^22^ and Aggour et al. (2020)^27^ had three unclear points, and the remaining three studies had two unclear points^23,26,28^.

### Study outcomes

#### Effect on ulcer severity

Table 2 presents detailed results on ulcer severity. All the included studies evaluated the severity of ulcers before and after treatment, but the specific content and methods of the assessment were not the same. Dugourd et al. (2020)^26^ determined the average decrease in canker sore counts, and the results were not significantly different between the two groups after 90 and 180 days. Mimura et al. (2017)^23^ determined the ulcer severity with the number of lesions and outbreaks, average healing time, and maximum lesion size. Whereas, probiotic treatment was not more effective in decreasing ulcer severity with 120 and 180 day follow-ups. Pedersen et al. (2019)^25^ estimated the ulcer severity score (USS) after 3 months, in which six characteristics (number, size, duration, ulcer-free period, site, and pain) were calculated to generate an ulcer score. Aggour et al. (2020) calculated the effectiveness indices (EI) of the ulcer size improvement. But in the two studies above, the difference was statistically insignificant between two groups. Trinchieri et al. (2011)^22^ calculated the total intensity score for all the patients in each group before and after treatment, reporting that *Lactobacillus brevis* CD2 lozenges were more effective in reducing ulcer severity compared with the placebo group. Different from studies above, Nirmala et al. (2017)^24^ and Aswath et al. (2014)^28^ applied probiotic as an adjunct and the results exhibiting more efficacy in reducing ulcer severity. It was impossible to perform a meta-analysis regarding the ulcer counts because of significantly different baseline characteristics, different interventions, and high heterogeneity.

**Table 2:** Outcomes related to ulcer severity in all the included studies.

Study ID	Evaluation index	Follow-up periods	Main outcome Mean (SD)		P value (T vs C)	Conclusion
			T	C (C_1_)		
Dugourd 2020^26^	Number of canker sores	Baseline	5.62 (2.61)	11.68 (8.66)	0.063	Not more effective compared with placebo group
		90 days	2.67 (2)	10.63 (14.68)	0.127	
		180 days	2.67 (1.87)	7.75 (7.87)	0.079	
Trinchieri 2011^22^	Intensity of Burning	Baseline	34	35	0.9	Significantly more effective compared with placebo group
		7 days	1	16	0.03	
	Intensity of Perilesional erythema	Baseline	34	34	NA	
		7 days	1	18	0.02	
	Sum of diameters of the greater individual ulcer	Baseline	112	121	NA	
		7 days	3	54	< 0.01	
Pedersen 2019^25^	USS	Baseline	30.1 (4.8)	25.1 (5.3)	NS	Not more effective compared with placebo group
		90 days	21.4 (7.7)	21.4 (8.1)	NS	
Nirmala 2017^24^	No. of ulcer	Baseline	1.6 (0.68)	1.65 (0.67)	0.816	Probiotic, as a local adjuvant to steroids, significantly reduces the ulcer severity compared with simple steroids group
		5 days	1.05 (0.88)	1.05 (0.94)	1	
		10 days	0.15 (0.37)	0.25 (0.44)	0.4422	
	Size of ulcer	Baseline	1.7 (0.86)	1.7 (0.8)	1	
		5 days	1.1 (0.71)	1.3 (0.8)	0.41	
		10 days	0.15 (0.37)	0.35 (0.59)	0.204	
	Erythema	Baseline	1.25 (0.44)	1.3 (0.8)	0.808	
		5 days	0.25 (0.44)	0.75 (0.44)	0.001	
		10 days	0 (0)	0.15 (0.37)	0.0749	
Mimura 2017^23^	Total number of lesions	Baseline	7.8 (7)	7.3 (4.5)	0.864	Not more effective compared with placebo group
		120 days	4.3 (4.7)	3.6 (3.6)		
		180 days	1.9 (1.8)	2.6 (2.1)		
	Average healing time (days)	Baseline	10.8 (5.9)	9.8 (3.9)	0.256	
		120 days	5.0 (3.1)	6.0 (5.5)		
		180 days	4.4 (3.6)	5.2 (3.4)		
	Maximum lesion size (mm)	Baseline	5.7 (3.4)	5.6 (1.6)	0.155	
		120 days	3.6 (2.7)	3.7 (2.9)		
		180 days	2.7 (2.1)	3.7 (2.4)		
	Number of outbreaks	Baseline	2.7 (1.7)	3.0 (1.5)	0.43	
		120 days	2.0 (1.5)	1.8 (1.6)		
		180 days	1.4 (1.6)	1.6 (1.6)		
Aggour 2020^27^	Effectiveness index (EI)	3 days	44.55% (20.79%)	31.66% (24.86%)	0.2149	Not more effective compared with Oracure gel group
		5 days	70.029% (15.17%)	62.76% (18.24%)	0.0891	
Aswath 2014^28^	No. of ulcers	Baseline	2.08 (1.89)	1.64 (1.08)	0.317	Significantly more effective compared with control group
		NR	0.46 (1.2)	0.48 (0.88)	0.904	
	Ulcer size	Baseline	4.80 (2.89)	5.44 (4.34)	0.542	
		NR	1.08 (2.42)	2.17 (4.49)	0.018	

*T* test group, *C* control group, *VAS* Visual Analogue Pain Scale, *USS* Ulcer Severity Score, *OHIP-14* Oral Health Impact Profile 14 questionnaire, *NS* not significant, *NA* not available, *NR* not reported.

#### Effect on oral pain

**Figure 3:**
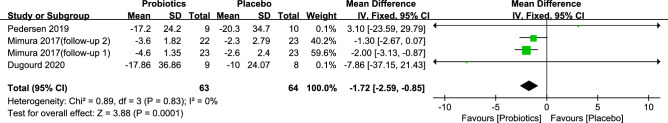
Forest plot for VAS scores regarding oral pain between probiotics and placebo.

Table 3 presents detailed results on oral pain. Six of the included studies used the effect on oral pain as an outcome indicator, but different assessment methods and follow-up periods were adopted. Three studies^23,25,26^ using VAS to evaluate oral pain related to the RAU were included in the meta-analysis, but all were described qualitatively below. Aggour et al. (2020) calculated the effectiveness indices (EI) of the pain improvement^27^. Nirmala et al. (2017) and Aggour et al. (2020)^24,27^ reported that probiotics, as a local adjunct, could significantly decrease oral pain compared with control group in the follow-up. With regard to the efficacy, four studies^22–24,27^ reported that probiotics exhibited a stronger capacity for reducing pain, while the other two^25,26^ found no significant differences between probiotics group and placebo group. A meta-analysis regarding oral pain was conducted. The inter-study heterogeneity regarding the oral pain related to RAU was low (*χ*^2^ = 0.83, *P* = 0.0001, *I*^2^ = 0%) and a fixed-effect model was selected. A higher decrease in oral pain was obtained in favor of probiotics treatment (*MD* = − 1.72, 95% *CI* [− 2.59; − 0.85], *P* = 0.07) (Fig. 3).

**Table 3:** Outcomes related to oral pain in all the included studies.

Study ID	Evaluation index	Follow-up periods	Main outcome Mean (SD)		P value (T vs C)	Conclusion
			T	C		
Dugourd 2020^26^	VAS	Baseline	52.33 (29.4)	48.88 (18.13)	0.778	Not more effective compared with placebo group
		180 days	41.71 (35.36)	38.88 (19.72)	0.848	
		180 days	14 (12.1)	12.38 (7.41)	0.756	
Trinchieri 2011^22^	The intensity of spontaneous pain	Baseline	33	34	NA	Significantly more effective compared with placebo group
		7 days	1	18	0.02	
	The intensity of caused pain	Baseline	39	40	NA	
		7 days	1	20	0.02	
Pedersen 2019^25^	VAS	Baseline	31.0 (13.9)	36.4 (26.8)	NS	Not more effective compared with placebo group
		90 days	13.8 (16.5)	13.6 (27.4)	NS	
Nirmala 2017^24^	Degree of pain	Baseline	2.95 (0.82)	2.65 (0.81)	0.253	Probiotic, as a local adjuvant to steroids, significantly reduces oral pain compared with simple steroids group
		5 days	0.95 (0.75)	2.0 (0.64)	0.0001	
		10 days	0 (0)	0.05 (0.22)	0.3236	
Mimura 2017^23^	VAS	Baseline	6.2 (1.1)	5.9 (1.9)	0.006	Significantly more effective compared with placebo group
		120 days	2.6 (2.1)	3.6 (3.2)		
		180 days	1.6 (1.5)	3.3 (2.7)		
Aggour 2020^27^	Effectiveness index (EI)	3 days	50.3% (18.85%)	40.67% (25.815%)	0.0455	Significantly more effective compared with Oracure gel group
		5 days	55.77% (26.131%)	44.51% (26.03%)	0.0093	

*T* test group, *C* control group, *VAS* Visual Analogue Pain Scale, *USS* Ulcer Severity Score, *OHIP-14* Oral Health Impact Profile 14 questionnaire, *NS* not significant, *NA* not available.

#### Quality of oral health

In addition to oral pain and the severity of the ulcer, Dugourd et al. (2020)^26^ assessed patients’ QoL in two groups before treatment and after 90 and 180 days with OHIP-14. The results showed no significant differences (*P* = 0.827).

#### Adverse effect

Mimura et al. (2017)^23^ observed flatulence and loose bowels during the symbiotic treatment, while no side effects were found in three studies (Trinchieri et al., 2011; Pederson et al., 2019; Dugourd et al., 2020)^22,25,26^. The remaining study did not report any side effects.

## Discussion

The present study assessed the efficacy of probiotics alone or as an adjunct to other drugs for recurrent aphthous stomatitis (RAS) patients compared with placebo or other drugs alone in terms of the severity of ulcers and oral pain. Only RCTs were included. The overall risk of bias in all the included studies was unclear based on the Cochrane risk-of-bias tool. Due to the high heterogeneity regarding the ulcer severity, a meta-analysis was not performed. The severity of ulcers was qualitatively analyzed, and a meta-analysis was carried out concerning oral pain. Generally, applying probiotics alone was effective in relieving oral pain but not effective in reducing ulcer severity. The results of the meta-analysis showed that higher oral pain relief was obtained in favor of probiotics treatment. Probiotics, as a local adjunct to steroids or anaesthetic antiseptic gel, could significantly reduce the ulcer severity and relieve oral pain. Overall, probiotics have a particular effect on RAS. However, there are still some points worth debate concerning (1) the dose and the application of mono versus mixed species probiotics therapy; (2) the outcome assessment measures and follow-up period; (3) the biosafety of probiotics; and (4) the quality of the included literature.

The ingredients and dosage of probiotics were not the same in the seven included studies. The probiotics ingredients in studies by Dugourd et al. (2020)^26^, Trinchieri et al. (2011)^22^, and Nirmala et al. (2017)^24^ consisted of *Lactobacillus rhamnosus* Lcr35, *Lactobacillus brevis* CD2, and *Bacillus clausii*, respectively. However, there were two probiotics (*Lactobacillus reuteri* DSM17938 and ATCC PTA5289) in Pedersen et al.’s study (2019)^25^ and four probiotics (*Lactobacillus paracasei* LPC-37 SD 5275, *Lactobacillus rhamnosus* HN001 SD 5675, *Lactobacillus acidophilus* NCFM SD 5221, *Bifidobacterium lactis* HN019 SD 5674) in Mimura et al.’s study (2017)^23^. Reham Lotfy Aggour et al.’s study (2020)^28^ contains *Lactobacillus acidophilus* and *Bifidobacterium lactis* and Nalini Aswath et al.’s study (2014)^27^ contains *Streptococcus faecalis* T-110, *Clostridium butyricum* TO-A, *Bacillus mesentericus* TO-A, *Lactobacillus sporogenes*. Four studies included in the present review used lozenges to administer probiotics in the treatment of aphthous ulcers^22,25,27,28^. Due to the availability of multiple probiotic strains that can be combined in different proportions, differences in probiotic ingredients and dosage between studies are currently inevitable. Six studies selected *Lactobacillus* for treatment^22,23,25–28^. Previous studies with dietary supplements containing *L. reuteri* have shown downregulation of proinflammatory cytokines and higher sIgA levels in the oral environment^29^. Dong et al. (2012)^30^ investigated the comparative effects of six probiotic strains on immune function in vitro and observed that all *Lactobacillus* strains induced the production of Th1 cytokines. *Lactobacillus* seems to be the ideal probiotic for the treatment of RAS. Nirmala et al. (2017)^24^ confirmed that the probiotic (*Bacillus clausii*) used as a local adjunct to steroids could significantly reduce the ulcer severity, which provided a new strategy for treating RAS. Moreover, in studies by Trinchieri et al. (2011)^23^ and Nirmala et al. (2017)^25^, *Lactobacillus brevis* CD2 and *Bacillus clausii* exhibited an excellent capacity for promoting ulcer healing and reducing oral pain. Different therapeutic effects might be related to different ingredients of probiotics. Future studies should further explore whether *Lactobacillus brevis* CD2 and *Bacillus clausii* can outperform other probiotics in treating RAS. The effects of different doses on therapeutic efficacy should also be explored.

As a useful tool for evaluating oral pain, VAS has exhibited high reliability and validity and is more objective than other evaluation methods included in the study. Various methods are available for evaluating the severity of ulcers. At this point, it was difficult to interpret and compare outcomes. Thus, a more standardized scoring system is required to assess the ulcer severity in RAS patients. Future studies also should aim to report on outcomes using a universally accepted evaluation index. The present study showed that the effect of probiotic treatment is closely related to the follow-up time. The follow-up periods of the studies by Trinchieri et al. (2011)^22^ and Nirmala et al. (2017)^24^ were 7 and 10 days, and the study carried by Aswath et al. (2014)^28^ and Aggour et al. (2020)^27^ last for 14 days and 5 days. The effects of probiotic therapy were more effective than the control group. However, for the other three studies, the follow-up periods were more than 90 days^23,25,26^. The results showed that they were not more effective in reducing ulcer severity and oral pain than the control group. In other fields, the duration of probiotic therapy and follow-up has been reported to be a confounder^31^. It can be speculated that probiotics are effective in treating aphthous ulcers in the short term, but the long-term effects are still to be elucidated, necessitating more high-quality studies.

No significant side effects were reported in the included studies. Besides, probiotics have exhibited biological safety in treating other diseases, and the safety of probiotic preparations is generally satisfactory. To date, the adverse effects of these probiotic drugs have been considered few, and reports on their harmful effects in the host are rare, except for some people with underlying medical conditions^32^. Currently, *Lactobacillus* and *Bifidobacterium* are effectively used to treat some intestinal diseases, such as diarrhea, which are almost the same as drugs used to treat some oral diseases^33,34^. Most probiotic products are marketed as food and given a designation called GRAS (generally regarded as safe)^35^. However, there may still be a systemic infection caused by bacterial translocation, excessive immune response, gastrointestinal adverse reactions, and other side effects^36,37^. Probiotic drugs used in patients with immune deficiency, severe diseases, and gastrointestinal epithelial injury are prone to systemic bacteremia and infection^38^. Probiotics might increase mortality in patients with severe acute pancreatitis^39^. *Lactobacillus* preparation might cause lactic acidosis and increase the risk of sepsis, meningitis, and other diseases^40^. Therefore, the application of probiotic preparations should be carefully observed to prevent disease progression. More studies are needed to elucidate the potential adverse effects of probiotic treatment.

All the included studies were RCTs, which ensured the credibility of the results. Also, we focused on applying probiotics in the treatment of oral ulcers, which might provide new ideas for treating oral ulcers in clinical practice. To the best of our knowledge, this article is the first systematic review to focus on the effect of probiotic drugs in RAS. However, this study had some drawbacks. Firstly, two studies (Dugourd et al., 2020, and Pedersen et al., 2019)^25,26^ only accounted for 0.1% of the weight in the meta-analysis, which should be attributed to the different sample size of the included study. Secondly, seven included studies all claimed double-blind use but six of the studies didn’t mention the technical details except Aggour et al. (2020)^27^, and only Mimura et al. (2017)^23^ mentioned that they used double-blind in participants and personnels. Thirdly, we still need more samples and studies to confirm the credibility of the results. Fourthly, patients’ oral hygiene status and microecological environment were not recorded, and whether the microbial distribution is correlated with the treatment effect is worth further exploration.

Due to the limited evidence of current studies, more high-quality studies with a better methodology are necessary before the final clinical application. A crossover design is considered more suitable for clinical trials on RAS, which is a recurrent, stable and agnogenic disease, than a parallel design. With this study design, on the one hand, every participant is compared to themselves, thus eliminating the influence of nonprocessing factors on the results, including sex, age, oral hygiene habits, oral health, general health, and smoking. If a parallel design is adopted, there should be no significant difference in these nonprocessing factors between the experimental and control groups. On the other hand, a smaller sample size is required to reach sufficient statistical power compared with parallel trials. A better study design is comprised of larger sample size, more reasonable randomization, more rigorous blinding, fewer missing outcome data, and more reasonable evaluation intervals, including short-term and long-term intervals. Currently, some clinical studies are underway, with only a few of them comparing the efficacy of probiotics with other therapeutic methods. Thus, future research should compare the effects of probiotics with mainstream drugs, such as steroids. Probiotics exhibited high efficacy in patients with low immunity ^41^. Clinical trials in such patients might have important implications. Finally, the relationship between RAS activity and critical bacteria should be investigated concerning the development and improvement of RAS.

## Methods

### Focused question

This systematic review was carried out following the Cochrane Handbook for the Systematic Reviews of Interventions and the preferred Reporting Items for Systematic Reviews and Meta-Analyses (PRISMA) guidelines^42^.

The focused PICO question was: Are probiotics alone or as an adjunct to other drugs more effective than placebo or other drugs alone in decreasing ulcer severity and oral pain in RAS patients?

The PICO principle was applied during the assessment.

Population/patient: Patients with recurrent aphthous ulcers (RAU).

Intervention: Treatment with probiotics, either alone or combined with other drugs.

Comparison: Treatment with placebo or other drugs alone.

Outcome: The severity of ulcer or oral pain.

### Selection criteria

The inclusion criteria consisted of (1) randomized controlled trials (RCT); (2) assessing the treatment of patients with recurrent aphthous ulcers; (3) comparing probiotics alone *versus* placebo or probiotics combined with other drugs versus other drugs alone.

The exclusion criteria consisted of (1) in vitro or animal studies, case reports, and case series, review articles, or opinion articles; (2) participants with any systematic disease or if they were duplicated or ancillary studies; (3) studies that did not include outcomes required in this review; and (4) studies that were still in process.

### Literature search

An electronic literature search was conducted in PubMed, EMBASE via OVID, the Cochrane Library (CENTRAL), and Web of Science scientific databases before September 2020 to identify relevant studies. Detailed search strategies in the four databases were shown in supplementary information.

After eliminating duplicates, two independent authors (L.S.Y. and C.B.) read the titles and abstracts derived from the electronic search to identify potentially eligible articles. If they conformed to the proposed theme, they were assessed in the full text and further evaluated for inclusion criteria. The references of all the included articles or relevant reviews were also considered to avoid missing any eligible studies. Any discrepancies between the authors were resolved by discussion.

### Data extraction

Three sheets were designed to extract the characteristics and numerical data of the included studies. Two researchers (L.S.Y. and C.B.) separately extracted the data, which included the following: study author, study design, the number of patients, mean age, the diagnosis of the patients, interventions, comparison, the number of patients who dropped out, evaluation index, evaluation interval, follow-up periods, primary outcomes, and conclusions. The evaluation parameters for the severity of ulcer and oral pain were reported using means standard deviations (SD), and *P*‐values. If data in the original articles were absent, the corresponding author was contacted for missing information. Any disagreement between researchers was resolved by discussion. If no agreement could be reached, a third researcher (Z.X.Y.) joined the discussion.

### Assessment of risk of bias and studies’ quality

The risk of bias was assessed according to the *Cochrane Collaboration’s tools*^43^. Two independent researchers conducted evaluations without interference. The main criteria for evaluation were the generation of random sequences, whether the assignment was hidden, whether the researchers and subjects were double-blinded, whether the study outcomes were blindly assessed, whether the results were complete, and whether the results were selectively reported. We also designed an additional risk of bias tables to assess other biases, such as whether the article-publishing organization was associated with medication manufacturers or not. Revman manager 5.3 was used for data visualization. If the two researchers’ assessments were not consistent, a third researcher reassessed the disputed results or conducted a team discussion.

### Statistical analyses

The level of heterogeneity was assessed by the Cochrane *χ*^2^ and *I*^2^ test. *P*-value of χ^2^ test < 0.1 or *I*^2^ > 50% was considered an indicator of high heterogeneity. *I*^2^ > 0.7 was considered too high to conduct a meta-analysis. The fixed-effect model was selected if *P* > 0.1 or *I*^2^ < 50%, while the random-effect model was selected if *P* < 0.1 or *I*^2^ > 50%. Mean difference (MD) and standard deviation (SD) were used to perform the meta-analysis. Weighted mean difference (WMD) and 95% confidence interval (CI) were selected as measure estimates of differences between the probiotic and placebo groups. All the statistical analyses were conducted using Review Manager 5.3.

## Conclusion

Probiotics alone were effective in relieving oral pain but not effective in reducing ulcer severity. The combination of probiotics and steroids or anaesthetic antiseptic gel was more effective than steroids or anaesthetic antiseptic gel alone in decreasing ulcer severity and oral pain for RAS patients. Probiotics are promising for the treatment of recurrent aphthous stomatitis. However, due to the limited data and study quality, more well-designed clinical studies are necessary.

## Supplementary information

Supplementary Tables.

## Data Availability

All the data were included in the paper and supplementary information.

## References

[1] Kleinman DV, Swango PA, Pindborg JJ (1994). Epidemiology of oral mucosal lesions in United States schoolchildren: 1986–87. Commun. Dent. Oral Epidemiol..

[2] Chiang CP, Chueh LH, Lin SK, Chen MY (1998). Oral manifestations of human immunodeficiency virus-infected patients in Taiwan. J. Formosan Med. Assoc..

[3] Koybasi S, Parlak AH, Serin E, Yilmaz F, Serin D (2006). Recurrent aphthous stomatitis: investigation of possible etiologic factors. Am. J. Otolaryngol..

[4] Cui RZ, Bruce AJ, Rogers RS (2016). Recurrent aphthous stomatitis. Clin. Dermatol..

[5] Baccaglini L (2011). Urban legends: recurrent aphthous stomatitis. Oral Dis..

[6] Schemel-Suárez M, López-López J, Chimenos-Küstner E (2015). Oral ulcers: differential diagnosis and treatment. Med. Clin. (Barc).

[7] Tarakji B, Gazal G, Al-Maweri SA, Azzeghaiby SN, Alaizari N (2015). Guideline for the diagnosis and treatment of recurrent aphthous stomatitis for dental practitioners. J. Int. Oral Health.

[8] Barile MF, Graykowski EA, Driscoll EJ, Riggs DB (1963). L form of bacteria isolated from recurrent aphthous stomatitis lesions. Oral Surg. Oral Med. Oral Pathol..

[9] Kim YJ (2016). Mucosal and salivary microbiota associated with recurrent aphthous stomatitis. BMC Microbiol..

[10] Slebioda Z, Szponar E, Kowalska A (2014). Etiopathogenesis of recurrent aphthous stomatitis and the role of immunologic aspects: literature review. Arch. Immunol. Ther. Exp. (Warsz).

[11] Belenguer-Guallar I, Jiménez-Soriano Y, Claramunt-Lozano A (2014). Treatment of recurrent aphthous stomatitis. A literature review. J. Clin. Exp. Dent..

[12] Altenburg A, Abdel-Naser MB, Seeber H, Abdallah M, Zouboulis CC (2007). Practical aspects of management of recurrent aphthous stomatitis. J. Eur. Acad. Dermatol. Venereol..

[13] Jinbu Y, Demitsu T (2014). Oral ulcerations due to drug medications. Jpn. Dent. Sci. Rev..

[14] Maldonado Galdeano C, Cazorla SI, Lemme Dumit JM, Vélez E, Perdigón G (2019). Beneficial effects of probiotic consumption on the immune system. Ann. Nutr. Metab..

[15] Hotel, A. Health and nutritional properties of probiotics in food including powder milk with live lactic acid bacteria–joint FAO/WHO Expert Consultation. **2014** (2001).

[16] Caglar E, Kargul B, Tanboga I (2005). Bacteriotherapy and probiotics' role on oral health. Oral Dis..

[17] Gruner D, Paris S, Schwendicke F (2016). Probiotics for managing caries and periodontitis: systematic review and meta-analysis. J. Dent..

[18] Khouly I, Pardiñas-López S, Ruff RR, Strauss FJ (2020). Efficacy of growth factors for the treatment of peri-implant diseases: a systematic review and meta-analysis. Clin. Oral Investig..

[19] Nguyen T (2020). Probiotics, including nisin-based probiotics, improve clinical and microbial outcomes relevant to oral and systemic diseases. Periodontology.

[20] Shu Z, Li P, Yu B, Huang S, Chen Y (2020). The effectiveness of probiotics in prevention and treatment of cancer therapy-induced oral mucositis: a systematic review and meta-analysis. Oral Oncol..

[21] Cappello F (2019). Probiotics can cure oral aphthous-like ulcers in inflammatory bowel disease patients: a review of the literature and a working hypothesis. Int. J. Mol. Sci..

[22] Trinchieri V, Carlo SD, Bossu M, Polimeni A (2011). Use of lozenges containing *Lactobacillus**brevis* CD2 in recurrent aphthous stomatitis: a double-blind placebo-controlled trial. Ulcers.

[23] Mimura MAM, Borra RC, Hirata CHW, de Oliveira Penido N (2017). Immune response of patients with recurrent aphthous stomatitis challenged with a symbiotic. J. Oral Pathol. Med..

[24] Nirmala M, Smitha SG, Kamath GJ (2017). A study to assess the efficacy of local application of oral probiotic in treating recurrent aphthous ulcer and oral candidiasis. Indian J. Otolaryngol. Head Neck Surg..

[25] Pedersen AML, Bukkehave KH, Bennett EP, Twetman S (2019). Effect of lozenges containing *Lactobacillus**reuteri* on the severity of recurrent aphthous ulcers: a pilot study. Probiotics Antimicrob. Proteins.

[26] Dugourd PM (2020). Probiotics for recurrent idiopathic aphthous stomatitis in adults: a placebo-controlled randomized trial. J. Eur. Acad. Dermatol. Venereol..

[27] Aggour RL, Mahmoud SH, Abdelwhab A (2020). Evaluation of the effect of probiotic lozenges in the treatment of recurrent aphthous stomatitis: a randomized, controlled clinical trial. Clin. Oral Investig..

[28] Aswath N, Kumar ST, Jayesh S, Manigandan T, Sarumathi T (2014). A randomized, open label, clinical study of synbiotics in patients with recurrent minor aphthous ulcers. Res. J. Pharm. Biol. Chem. Sci..

[29] Braathen G, Ingildsen V, Twetman S, Ericson D, Jørgensen MR (2017). Presence of *Lactobacillus**reuteri* in saliva coincide with higher salivary IgA in young adults after intake of probiotic lozenges. Benef. Microbes.

[30] Dong H, Rowland I, Yaqoob P (2012). Comparative effects of six probiotic strains on immune function in vitro. Br. J. Nutr..

[31] Elazab N (2013). Probiotic administration in early life, atopy, and asthma: a meta-analysis of clinical trials. Pediatrics.

[32] Sanders ME (2010). Safety assessment of probiotics for human use. Gut Microbes.

[33] Bizzini B, Pizzo G, Scapagnini G, Nuzzo D, Vasto S (2012). Probiotics and oral health. Curr. Pharm. Des..

[34] Kumar AV (2013). Probiotics: nature's medicine. Int. J. Nutr. Pharmacol. Neurol. Dis..

[35] Liong MT (2008). Safety of probiotics: translocation and infection. Nutr. Rev..

[36] Doron S, Snydman DR (2015). Risk and safety of probiotics. Clin. Infect. Dis..

[37] Seale JV, Millar M (2013). Probiotics: a new frontier for infection control. J. Hosp. Infect..

[38] Expression of concern—probiotic prophylaxis in predicted severe acute pancreatitis: a randomised, double-blind, placebo-controlled trial. *Lancet *(*London, England*) **375**, 875–876. 10.1016/s0140-6736(10)60360-1 (2010). 10.1016/S0140-6736(10)60360-1 20226971

[39] Elias J, Bozzo P, Einarson A (2011). Are probiotics safe for use during pregnancy and lactation?. Can. Fam. Physician.

[40] Cruchet S (2015). The use of probiotics in pediatric gastroenterology: a review of the literature and recommendations by Latin-American experts. Paediatr. Drugs.

[41] Niscola P (2012). Aphthous oral ulceration and its successful management by *Lactobacillus**brevis* CD2 extract in an adult haemophilic patient. Haemophilia.

[42] Moher D (2015). Preferred reporting items for systematic review and meta-analysis protocols (PRISMA-P) 2015 statement. Syst. Rev..

[43] Higgins JP (2011). The Cochrane Collaboration's tool for assessing risk of bias in randomised trials. BMJ.

