# Supplementary material for: The efficacy of probiotics in management of recurrent aphthous stomatitis: a systematic review and meta-analysis
Source: Sci Rep. 2020 Dec 3;10:21181. doi: 10.1038/s41598-020-78281-7 (PMC7713296; doi:10.1038/s41598-020-78281-7)
Supplement: Supplementary file 1 — Supplementary Tables. [file 41598_2020_78281_MOESM1_ESM.docx]

**The efficacy of probiotics in management of recurrent aphthous stomatitis: a systematic review and meta-analysis**

Bin Cheng1*, Xinyi Zeng1*, Shaoyuan Liu 1*, Jing Zou1, Yan Wang1#

*These authors contributed equally to the study and should be regarded as co-first authors.

1 State Key Laboratory of Oral Diseases, National Clinical Research Center for Oral Diseases & Dept. of Pediatric Dentistry, West China Hospital of Stomatology, Sichuan University, Chengdu 610041, China

**Supplementary Table S1.** Search strategy for Pubmed

**Supplementary Table S2.** Search strategy for embase through OVID

| #1 | [Mesh]probiotics explode all trees |
| --- | --- |
| #2 | [Mesh]Lactobacillales explode all trees |
| #3 | [Mesh]Lactococcus explode all trees |
| #4 | [Mesh]Bifidobacterium explode all trees |
| #5 | #1 OR #2 OR #3 OR #4 |
| #6 | probiotics[Title/Abstract] OR probiotic[Title/Abstract] OR lactobacilli[Title/Abstract] OR Lactobacillales[Title/Abstract] OR Lactococcus[Title/Abstract] OR Bifidobacterium[Title/Abstract] OR Bifidobacteria[Title/Abstract] |
| #7 | #5 OR #6 |
| #8 | aphthous[Title/Abstract] OR aphtha[Title/Abstract] OR canker sore[Title/Abstract] OR oral ulcer[Title/Abstract] |
| #9 | [Mesh]stomatitis, aphthous explode all trees |
| #10 | [Mesh]oral ulcer explode all trees |
| #11 | #9 OR #10 |
| #12 | #8 OR #11 |
| #13 | (#7 AND #12) |

| #1 | (aphthous OR aphtha OR canker sore OR oral ulcer).ab. |
| --- | --- |
| #2 | (Probiotics OR Probiotic OR Lactobacilli OR Lactobacillales or Lactococcus OR Bifidobacterium OR Bifidobacteria).ab. |
| #3 | #1 AND #2 |

**Supplementary Table S3.** Search strategy for Web of Science

| #1 | [topic]aphthous OR [topic]aphtha OR [topic]canker sore OR [topic]oral ulcer |
| --- | --- |
| #2 | [topic]Probiotics OR Probiotic OR [topic]Lactobacilli OR [topic]Lactobacillales OR [topic]Lactococcus OR [topic]Bifidobacterium OR [topic]Bifidobacteria |
| #3 | #1 AND #2 |

**Supplementary Table S4.** Search strategy for Cochrane Library

| #1 | [Mesh]probiotics explode all trees |
| --- | --- |
| #2 | [Mesh]Lactobacillales explode all trees |
| #3 | [Mesh]Lactococcus explode all trees |
| #4 | [Mesh]Bifidobacterium explode all trees |
| #5 | #1 OR #2 OR #3 OR #4 |
| #6 | [ti, ab, kw] probiotic* OR [ti, ab, kw] lactobacill* OR [ti, ab, kw]lactococcus* OR [ti, ab, kw] bifidobacteri* |
| #7 | #5 OR #6 |
| #8 | [Mesh]stomatitis, aphthous explode all trees |
| #9 | [Mesh]oral ulcer explode all trees |
| #10 | #8 OR #9 |
| #11 | [ti, ab, kw]aphthous OR [ti, ab, kw]aphtha OR [ti, ab, kw]canker sore OR [ti, ab, kw]oral ulcer |
| #12 | #10 OR #11 |
| #13 | #7 AND #12 |
